# Declining Levels of Neutralizing Antibodies Against SARS-CoV-2 in Convalescent COVID-19 Patients One Year Post Symptom Onset

**DOI:** 10.3389/fimmu.2021.708523

**Published:** 2021-06-16

**Authors:** Tiandan Xiang, Boyun Liang, Yaohui Fang, Sihong Lu, Sumeng Li, Hua Wang, Huadong Li, Xiaoli Yang, Shu Shen, Bin Zhu, Baoju Wang, Jun Wu, Jia Liu, Mengji Lu, Dongliang Yang, Ulf Dittmer, Mirko Trilling, Fei Deng, Xin Zheng

**Affiliations:** ^1^ Department of Infectious Diseases, Union Hospital, Tongji Medical College, Huazhong University of Science and Technology, Wuhan, China; ^2^ Joint International Laboratory of Infection and Immunity, Huazhong University of Science and Technology, Wuhan, China; ^3^ State Key Laboratory of Virology, Wuhan Institute of Virology, Chinese Academy of Sciences, Wuhan, China; ^4^ Institute for Virology, University Hospital of Essen, University of Duisburg-Essen, Essen, Germany

**Keywords:** SARS-CoV-2, COVID-19, spike, antibody responses, humoral immunity

## Abstract

Major advances have been made in understanding the dynamics of humoral immunity briefly after the acute coronavirus disease 2019 (COVID-19). However, knowledge concerning long-term kinetics of antibody responses in convalescent patients is limited. During a one-year period post symptom onset, we longitudinally collected 162 samples from 76 patients and quantified IgM and IgG antibodies recognizing the nucleocapsid (N) protein or the receptor binding domain (RBD) of the spike protein (S). After one year, approximately 90% of recovered patients still had detectable SARS-CoV-2-specific IgG antibodies recognizing N and RBD-S. Intriguingly, neutralizing activity was only detectable in ~43% of patients. When neutralization tests against the E484K-mutated variant of concern (VOC) B.1.351 (initially identified in South Africa) were performed among patients who neutralize the original virus, the capacity to neutralize was even further diminished to 22.6% of donors. Despite declining N- and S-specific IgG titers, a considerable fraction of recovered patients had detectable neutralizing activity one year after infection. However, neutralizing capacities, in particular against an E484K-mutated VOC were only detectable in a minority of patients one year after symptomatic COVID-19. Our findings shed light on the kinetics of long-term immune responses after natural SARS-CoV-2 infection and argue for vaccinations of individuals who experienced a natural infection to protect against emerging VOC.

## Introduction

*Coronavirus disease 2019* (COVID-19) caused by the novel *severe acute respiratory syndrome coronavirus 2* (SARS-CoV-2) currently causes a global pandemic with more than 3.48 million fatalities so far. Clinical manifestations of COVID-19 range from asymptomatic and mild infections to life-threatening pneumonia. The latter can only be survived with respiratory ventilation support (1, 2). SARS-CoV-2 particles contain the four main structural proteins spike (S), membrane (M), envelope (E), and nucleocapsid (N) protein (3, 4). The receptor binding domain (RBD) of the S protein binds tightly to the human *angiotensin-converting enzyme 2* (ACE2), initiating virus entry into host cells (5, 6). Hence, the S protein is regarded as the most relevant antigen eliciting crucial antibody responses in terms of protection. Accordingly, most SARS-CoV-2 vaccines aim to induce sustained S-specific IgG responses in order to mount potent neutralizing antibody responses (7), which are considered to represent correlates of protection.

Humoral immune responses constitute an indispensable part of adaptive immunity against various viral diseases (8). Several studies showed that most COVID-19 patients raise detectable SARS-CoV-2-specific antibodies recognizing the N protein and the RBD of the S protein during acute and early convalescent phases (9–11). We and others provided ample evidence that the occurrence and sustainability of SARS-CoV-2-specific antibodies is associated with the occurrence and severity of symptoms during the early phase directly after infection (12–15). Accordingly, COVID-19 patients with very mild or asymptomatic infection show a more rapid decay of antibody levels during the first months of recovery (16, 17), while recent studies indicate that antibody titers in convalescent patients who experienced more noticeable symptoms are stable for at least 6-9 months (18–21). In this context, it needs to be highlighted that the aforementioned duration was merely defined by the end of the conducted studies rather than a complete disappearance of antibodies. Nevertheless, little is known about the long-term durability of SARS-CoV-2-specific IgG and neutralizing antibody (nAb) responses following symptomatic infection. An understanding of the kinetics of waning immunity and the residual magnitude of antibody responses following natural SARS-CoV-2 infection is crucial for decision-making in terms of global vaccine programmes and mitigation strategies.

Recently, novel SARS-CoV-2 variants of concerns (VOC) such as the B.1.1.7, B.1.351, and P.1 lineage were identified in UK, Brazil, and South Africa, respectively (22–24). Obviously, the immediate question arose, if convalescent plasma (CP) obtained from individuals after natural infection possess the capacity to neutralize such emerging VOC (25). In particular the amino acid exchange E484K in the S protein, e.g. present in the B.1.351 lineage, has been shown to confer significant but incomplete immune evasive capacities by causing partial resistance to certain monoclonal antibodies, CP, and a fraction of post-vaccination sera (24, 26–28). The ability of the virus to circumvent parts of the protective immunity threatens the protection mediated by natural infections and current vaccines. In regions in which the viral spread was virtually terminated such as parts of China, re-exposure to emerging VOC fortunately did not occur so far. This prevented the generation of VOC-specific immune responses, raising the important question how well citizens would be protected if VOCs might be inadvertently introduced into the population. To address this relevant issue, we quantified the titers of SARS-CoV-2-specific IgM and IgG antibodies binding to the RBD of the S protein (Anti-S IgM/Anti-S IgG) or N protein (Anti-N IgM/Anti-N IgG) during a one-year period following symptom onset. Furthermore, we determined the neutralizing activity against the original SARS-CoV-2 that had emerged in 2019, for convenience denoted ‘wild type’ (WT) here, and the VOC B.1.351.

## Methods

### Participants and Data Collection

To study the sustainability of SARS-CoV-2-specific antibodies, we recruited 76 of the very first COVID-19 patients who were infected during the period between December 2019 and March 2020. At this time, the patients had been hospitalized at the Wuhan Union Hospital (a designed hospital for patients with COVID-19). All patients were followed up longitudinally for one year after the onset of symptoms. Clinical data including demographics, clinical manifestations, and comorbidities during the acute phase were obtained from electronic medical records supplemented by data collection through standardized questionnaires conducted by trained medical workers.

We included COVID-19 patients who had a unanimous clinical diagnosis or a laboratory diagnosis such as a positive RT-PCR test. All patients had at least one positive IgG antibody test during the hospitalization or follow-up period. We excluded the following patients: (I) those with fever, runny nose, cough, and other symptoms of upper respiratory tract infections experienced within one month of the follow-up, (II) those with acquired immunodeficiency disease syndrome due to an HIV infection, (III) patients suffering from autoimmune disease taking oral hormonal or immunosuppressive drugs, (IV) those who have received convalescent plasma therapy, (V) those who have been vaccinated against COVID-19, and (VI) pregnant women.

According to the Guidelines of the Diagnosis and Treatment for SARS-CoV-2 issued by the Chinese National Health Committee (Version 7), mild symptoms were defined as follows: (I) epidemiological history, (II) fever or other respiratory symptoms, (III) typical CT image abnormities indicating viral pneumonia, and/or decreased lymphocyte counts, (IV) positive result of a RT-PCR for SARS-CoV-2 RNA and/or positive result in a SARS-CoV-2-specific serologic IgG or IgM test. Severe symptoms additionally met at least one of the following conditions: (I) low oxygen saturation (≤93%) at resting state and/or PaO_2_/FiO_2_ ≤ 300mmHg, (II) respiratory failure and/or need for mechanical ventilation (III), multiple organ failure and/or admittance to an ICU. Accordingly, disease severity was defined based on above-mentioned criteria.

In order to further study the neutralizing capacity of convalescent plasma against the E484K-mutated VOC, we collected 53 samples during a 6 months period after infection to assess the neutralizing activity. Nineteen of the samples were derived from the aforementioned cohort.

### Sample Collection and Isolation

Five mL of heparin sodium anticoagulated venous blood from participants were collected to isolate plasma. All the samples were processed within the first 4h after collection. After centrifugation at 3000rpm for 15 min, plasma samples were separated and stored at -80°C for further experiments.

### CLIA-Based Detection of Binding Antibodies Recognizing SARS-CoV-2 S/N

As reported previously (29, 30), specific IgM and IgG antibodies recognizing the RBD of S the protein or the N protein were quantified using *capture chemiluminescence immunoassays* (CLIA) by MAGLUMI™ 4000 Plus (Snibe, Shenzhen, China). The cut-off value was 0.7 AU/mL for anti-S IgM and 1.0 AU/mL for anti-N IgM, anti-S IgG, and anti-N IgG. The sensitivity and specificity of CLIA-based detection are documented in **Table S1**.

### Virus Neutralization Test (VNT) Assays

The method of virus neutralization test has been described in our previous studies (30). Briefly, patient plasma was heated at 56°C for 30 minutes to inactivate the complement. Subsequently, plasma samples were serially diluted (two-fold dilutions) with Eagle’s Minimal Essential Medium (EMEM) (NewZongke, Wuhan, China) containing 2% (v/v) foetal bovine serum (FBS) (Gibco, CA, USA). SARS-CoV-2 suspensions (Strain BetaCoV/Wuhan/WIV04/2019, National Virus Resource Center number: IVCAS 6.7512 or the VOC B.1.351 (initially identified in South Africa) at 100 TCID_50_ were incubated with diluted plasma at 37°C for 1 h. Afterwards, Vero E6 cells (1*10^4^ per well) were overlaid with plasma-virus suspensions. Each neutralization test was performed in triplicates. At 48 h post-infection, virus-specific cytopathic effects (CPE) were visualized and judged by light microscopy. Neutralizing antibody titers are expressed as reciprocal values of the highest dilution preventing CPE formation.

### Statistical Analysis

Categorical variables are expressed as percentage, and significance was calculated by the chi-square or Fisher’s exact test. Continuous variables are expressed as mean ± SD or median ± 95% CI as appropriate, and significance was calculated applying two-tailed unpaired t test, one-way ANOVA, Mann-Whitney U test or Wilcoxon test as appropriate. SPSS (version 25, IBM, USA) and GraphPad Prism (version 8.0, San Diego, California, USA) software were applied for statistical analysis. In all statistical analyses, p <0.05 was considered to be statistically significant (ns: no significance; *p<0.05; **p<0.01; ***p<0.001; ****p<0.0001).

## Results

### Characterization of Patients at Baseline

A total of 162 serum samples from 76 SARS-CoV-2-infected individuals including some of the very earliest COVID-19 patients were collected in our study. Given that nucleic acid tests were not available during the initial COVID-19 outbreak, 28 (36.8%) patients were diagnosed based on prototypical clinical features as outlined in the methods section. Forty-eight (63.2%) patients were laboratory-confirmed by a positive RT-PCR test. All patients had at least one unanimous antibody test. Baseline data such as demographic and clinical characteristics of all participants are shown in **Table 1**. Among the 76 patients, 23 experienced severe and 53 non-severe courses of acute infection (see methods section for clinical definition criteria). The median age was 60 (46.5-67.0) years. Thirty-five patients (46.1%) were males. The most common symptom during hospitalization was fever (55 patients, 72.4%), followed by (dry) cough (43 patients, 56.6%), and fatigue (31 patients, 40.8%). Forty-three percent of individuals had pre-existing diseases. In this regard, hypertension and diabetes were the most common comorbidities (**Table 1**).

**Table 1:** Clinical characteristics of enrolled patients.

Characteristics	Total (N=76)	Severe (N=23)	Non-severe (N=53)	*P value*
Time from symptom onset to follow-up, Days, [median (IQR)]	350.5 (330.3-358.8)	346 (329-356)	352 (331-359)	0.277
Age, [median (IQR)]	60 (46.5-67.0)	57 (46-63)	62 (48-68)	0.818
Sex, Male [n (%)]	35 (46.1%)	11(47.8%)	24 (45.3%)	0.838
**Signs and symptoms at admission [n (%)]**				
Fever	55 (72.4%)	20 (87.0%)	35 (66.0%)	0.061
Chill	14 (18.4%)	5 (21.7%)	9 (17.0%)	0.739
Fatigue	31 (40.8%)	7 (30.4%)	24 (45.3%)	0.226
Headache	4 (5.3%)	1 (4.3%)	3 (5.7%)	>0.999
Myalgia	12 (15.8%)	1 (4.3%)	11 (20.8%)	0.093
Cough	43 (56.6%)	14 (60.9%)	29 (54.7%)	0.619
Chest tightness	11 (14.5%)	5 (21.7%)	6 (11.3%)	0.292
Chest pain	1 (1.3%)	0 (0%)	1 (1.9%)	>0.999
Shortness of breath	11 (14.5%)	3 (13.0%)	8 (15.1%)	>0.999
Dyspnea	6 (7.9%)	1 (4.3%)	5 (9.4%)	0.661
Loss of appetite	12 (15.8%)	4 (17.4%)	8 (15.1%)	>0.999
Nausea	2 (2.6%)	0 (0%)	2 (3.8%)	>0.999
Vomiting	2 (2.6%)	1 (4.3%)	1 (1.9%)	0.516
Abdominal pain	2 (2.6%)	0 (0%)	2 (3.8%)	>0.999
Diarrhea	8 (10.5%)	2 (8.7%)	6 (11.3%)	>0.999
**Comorbidities [n (%)]**				
Diabetes	11 (14.5%)	4 (17.4%)	7 (13.2%)	0.726
Hypertension	22 (28.9%)	7 (30.4%)	15 (28.3%)	0.851
CVD	2 (2.6%)	0 (0%)	2 (3.8%)	>0.999
COPD	1 (1.3%)	1 (4.3%)	0 (0%)	0.303

CVD, Cardiovascular Disease; COPD, chronic obstructive pulmonary disease.

P values indicate differences between severe and non-severe patients.

P < 0.05 was considered statistically significant.

Statistically significant was tested using Student’s t test, Chi-square test or Fisher exact test, depending on the applicability.

### SARS-CoV-2-Specific IgG Immune Responses Wane Over Time but Remain Positive During the First Year After Infection

**Figure 1:**
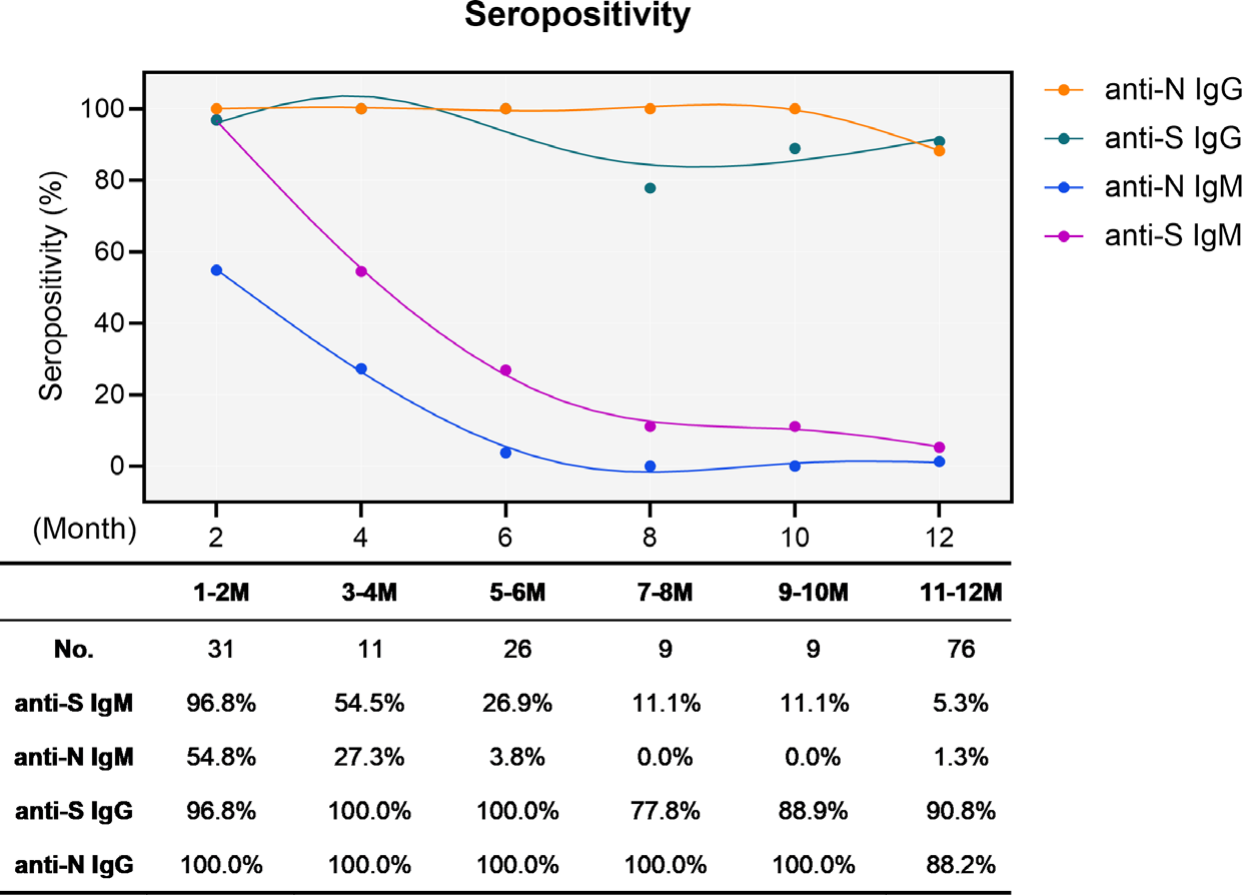
Sero-positivity of virus-specific IgG and IgM antibodies in 76 convalescent COVID-19 patients over time. The x-axis indicates the timeline following the onset of symptoms. Curves show the overall sero-positivity of IgG and IgM antibodies recognizing the RBD of the spike protein (anti-S IgG: green; anti-S IgM: purple) and the nucleoprotein (anti-N IgG: orange; anti-N IgM: blue). Numbers of samples (No.) at different time points are depicted under the diagram.

We first determined the sero-positivity concerning IgM and IgG either recognizing the N protein or the RBD of S protein. As expected, the S- and N-specific IgM started at intermediate to high levels (96.8 and 54.8%, respectively) early after infection and rapidly and dramatically waned over time. At the end of the one-year observation period, only four cases remained positive for both anti-S and anti-N IgM, resulting in residual positivity rates of 5.3% and 1.3%, respectively (**Figure 1**). Conversely, the overall sero-positivity for IgG antibodies in convalescent individuals remained very stable with 90.8% and 88.2% sero-positivity for anti-S and anti-N IgG, respectively, persisting for one year (**Figure 1**).

**Figure 2:**
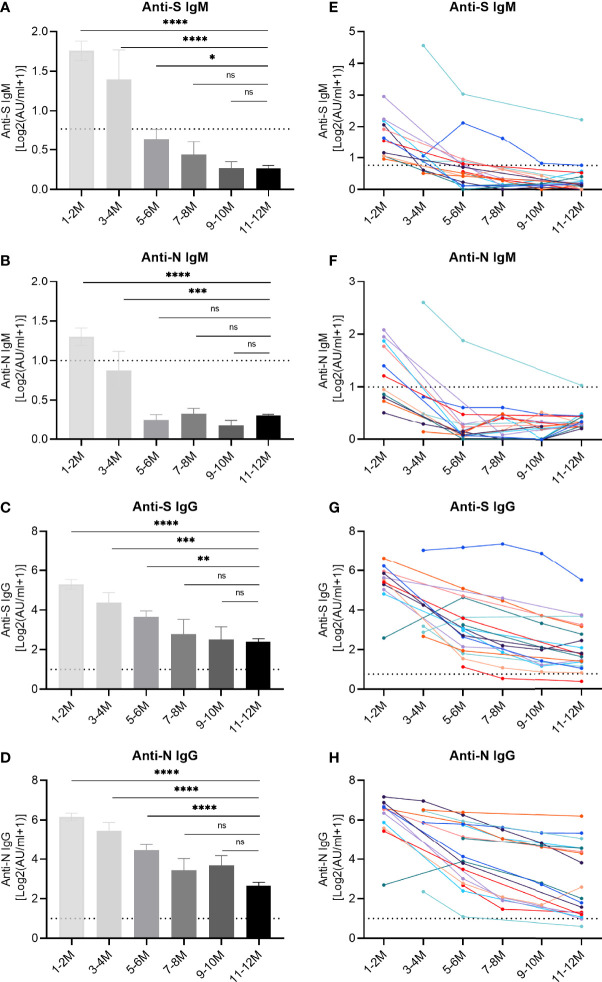
Longitudinal IgM and IgG antibody responses recognizing different SARS-CoV-2 proteins. IgM and IgG recognizing the RBD of the spike protein (S) or the nucleoprotein (N) were quantified by capture chemiluminescence immunoassays (CLIA) in 162 samples derived from 76 convalescent patients. **(A–D)** The plasma antibody levels of anti-S IgM, anti-N IgM, anti-S IgG, and anti-N IgG in convalescent patients. The numbers of samples at different time points were as follows: 31 (1-2 month), 11 (3-4 month), 26 (5-6 month), 9 (7-8 month), 9 (9-10 month), and 76 (11-12 month). **(E–H)** Repetitive sampling of 18 convalescent patients confirmed the kinetics of IgM and IgG responses recognizing SARS-CoV-2. The horizontal dotted line represents the cut-off value: 0.7 AU/mL (anti-IgM-S) and 1.0 AU/mL (anti-IgM-N, anti-IgG-S, and anti-IgG-N). P values were determined using a one-way ANOVA test. ns, no significance; *p < 0.05; **p < 0.01; ***p < 0.001; ****p < 0.0001.

Beyond the simple positivity above the detection limit, we further evaluated the dynamics of specific antibody titers (**Figure 2**). The titers of anti-S IgM and anti-N IgM showed a peak during the first period, as determined one to two months post symptom onset, and decreased over time. IgM responses dropped below the cut-off value after 5-6 months (**Figures 2A, B**), while the IgG titers continuously declined but mostly remained clearly above the detection limit (**Figures 2C, D**). At the end, the decrease in titers stopped and no significant difference was observed for IgG recognizing the RBD of the S protein and the N protein, when we compared the periods 9-10 and 11-12 month post symptom onset with each other (**Figures 2C, D**), suggesting a slowing down of the decay rate at later stages. These findings were corroborated when longitudinal kinetics of 18 individual patients were assessed (**Figures 2E–H**). In order to highlight the declining trends of antibodies, average antibody titers at different longitudinal time points are shown in the supplementary material (**Figure S1**).

**Figure 3:**
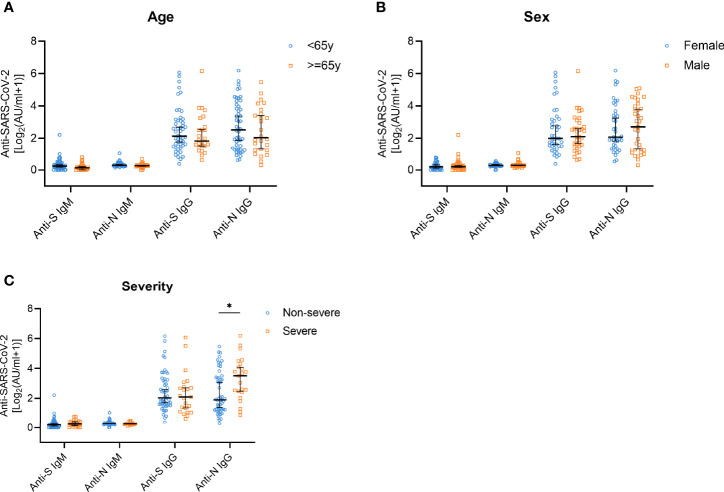
Anti-S/N IgM and anti-S/N IgG antibody responses on year after disease onset in patients stratified according to age, sex, and disease severity. **(A)** Comparison of anti-S IgM, anti-N IgM, anti-S IgG, and anti-N IgG titers in patients stratified according to age; < 65 years (n=50, blue) and ≥ 65 years (n=26, yellow). **(B)** Comparison of anti-S IgM, anti-N IgM, anti-S IgG, and anti-N IgG titers in patients grouped according to sex; female (n=41, blue) and male (n=35, yellow). **(C)** Comparison of anti-S IgM, anti-N IgM, anti-S IgG, and anti-N IgG titers in patients stratified according to disease severity; non-severe (n=53, blue) and severe (n=23, yellow). Data are presented as median ± 95% CI. *P* values were determined applying a two-tailed Mann-Whitney U test. *p < 0.05.

To study the changes of antibody titers according to age, sex, and disease severity, we performed analyses of stratified subgroups. The SARS-CoV-2 antibody levels did not show significant changes depending on age and sex (**Figures 3A, B**). However, anti-N IgG antibody titers in the group of people who experienced severe symptoms were significantly higher compared with the group with non-severe symptoms (*p*=0.013). No such differences were observed for the IgM antibodies or anti-S IgG levels (**Figure 3C**).

### SARS-CoV-2-Specific Neutralizing Antibodies Wane Considerably During the First Year After Infection

**Figure 4:**
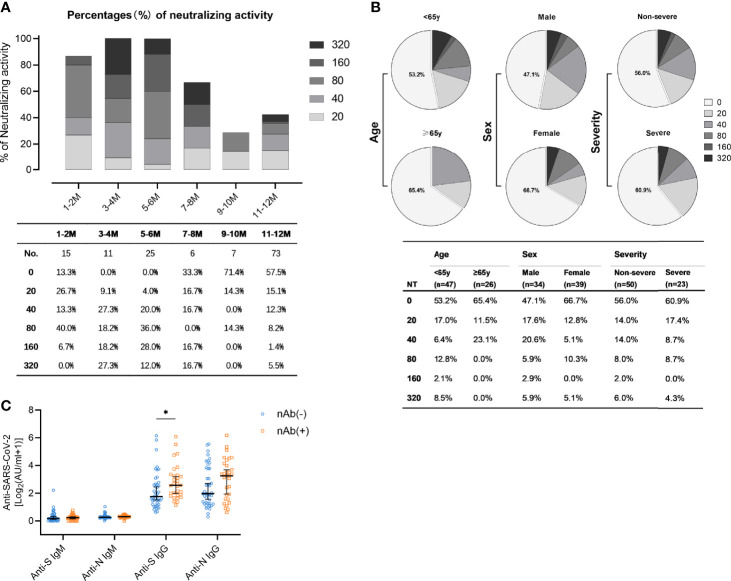
Levels of neutralizing activity in convalescent COVID-19 patients. **(A)** The neutralizing activity was quantified in 137 samples obtained from 76 patients. Different colored boxes depict indicated SARS-CoV-2 neutralization titers at indicated times after symptom onset. Numbers of samples (No.) at different time points are shown below the diagram. **(B)** Neutralization activity one year post symptom onset in patients stratified according to age (upper: <65y, lower: ≥65y), sex (upper: male, lower: female), and disease severity (upper: non-severe, lower: severe). **(C)** Comparison of anti-S IgM, anti-N IgM, anti-S IgG, and anti-N IgG in the nAb-positive (n=31, yellow) and the nAb-negative population (n=42, blue) one year post symptom onset. Data are presented as median ± 95% CI. *P* values were determined with a two-tailed Mann-Whitney U test. *p < 0.05.

Among all antibodies, those preventing the infection by neutralizing the incoming virus are considered to represent the most important ones. Accordingly, nAbs are regarded as relevant correlate of protection. SARS-CoV-2-specific neutralizing activity was measured for 73 patients by virus neutralization assays. The results showed that the majority of patients (57.5%) did not exhibit detectable neutralization capacities one year after the symptom onset (**Figure 4**). The proportion of patients with high titers of neutralizing antibodies (defined by titers exceeding 1:160) was highest 3-4 months after symptom onset (**Figure 4A**). Among the minority of patients who still had detectable nAb (42.5%), most individuals showed rather low nAb titers (≤1:80). Only very few (5.5%) patients exhibited strong neutralizing titers at or above 1:320 (**Figure 4A**). Subgroup analyses were also performed to compare neutralizing activities in convalescent patients with different age, sex, and disease severity (**Figure 4B**). Males, people younger than 65 years, and non-severe patients had a higher proportion of neutralizing antibodies. Neutralization titers exceeding 1:20 were detected in 39.1% (9/23) of patients with a severe course of disease and 44% (22/50) of the non-severe courses (**Figure 4B**). In accordance with the fact that S is the target of nAbs, anti-S IgG antibody titers in nAb-positive patients were significantly more frequent than among nAb-negative patients (**Figure 4C**, *p*=0.012). As expected and consistent with their negligible role for true complement-independent neutralization, there was no significant difference in the titers of anti-N IgG, anti-N IgM, and anti-S IgM between the population harbouring nAbs compared to the population lacking detectable nAbs (**Figure 4C**).

**Figure 5:**
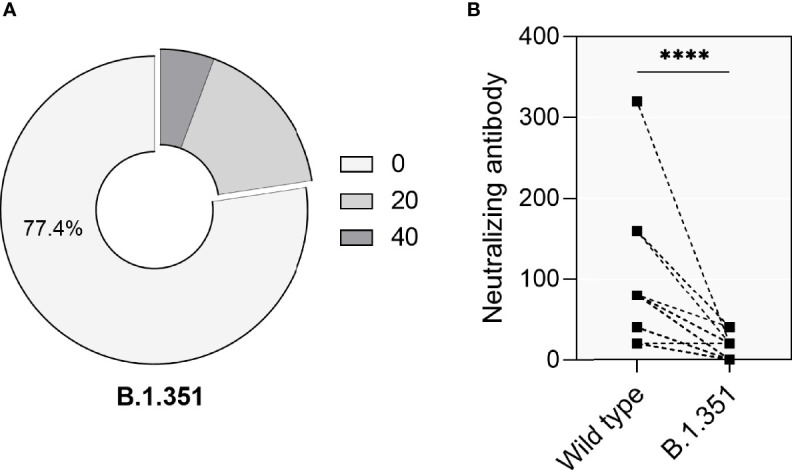
Neutralizing activity of 53 convalescent patients against the VOC B.1. 351. **(A)** The percentage of convalescent patients showing neutralizing activity against the VOC B.1.351. **(B)** Direct comparison of neutralization titers against the original SARS-CoV-2 strains (‘Wild type’) and VOC B.1.351 in 53 convalescent patients. P values were determined using a two-tailed Wilcoxon test. ns, no significance; ****p < 0.0001.

Given the emergence of VOCs and the discussion concerning their immune evasion capacity and their potential to cause reinfection events after immunity had waned over time, we further assessed the neutralization capacity against the VOC B.1.351 using sera collected during a 6 months period after infection from 53 convalescent patients which showed sustained neutralization activity against wt SARS-CoV-2. These patients comprised 25 males and 28 females, with a medium age 58 ± 14 years. Only 12 (22.6%) patients showed neutralizing activity against B.1.351, of which 9 (17.0%) cases had neutralizing titers of 1:20 and 3 (5.7%) patients had neutralizing titers of 1:40 (**Figure 5A**). The direct comparison of antibodies either capable to neutralize wt- or B.1.351 showed that neutralization titers against the VOC had decreased significantly (**Figure 5B**, *p*<0.0001).

## Discussion

One of the critical issues in the ongoing COVID-19 pandemic is to understand the magnitude and kinetics of the protective humoral immunity to SARS-CoV-2 following natural infection.

In this study, we characterized the sustainability of SARS-CoV-2-specific IgG and IgM antibody responses in convalescent COVID-19 patients in a group that, to our knowledge, represents the cohort with the longest follow-up period worldwide. One year after symptom onset, approximately 90% of patients showed detectable SARS-CoV-2-specific IgG antibodies above the limit of detection, while only very few maintained their IgM sero-positivity. However, only 43% percent of the patients had neutralizing activity after this long period. Among these individuals with significant neutralization activity against wt-SARS-CoV-2, even fewer (~22%) were capable to neutralize the VOC B.1.351. Thus, when re-exposure does not occur, and humoral immunity wanes over time, VOC harbouring immune evasive capacities pose a particular danger for populations that acquired their SARS-CoV-2-specific immunity through natural infection.

**Figure 6:**
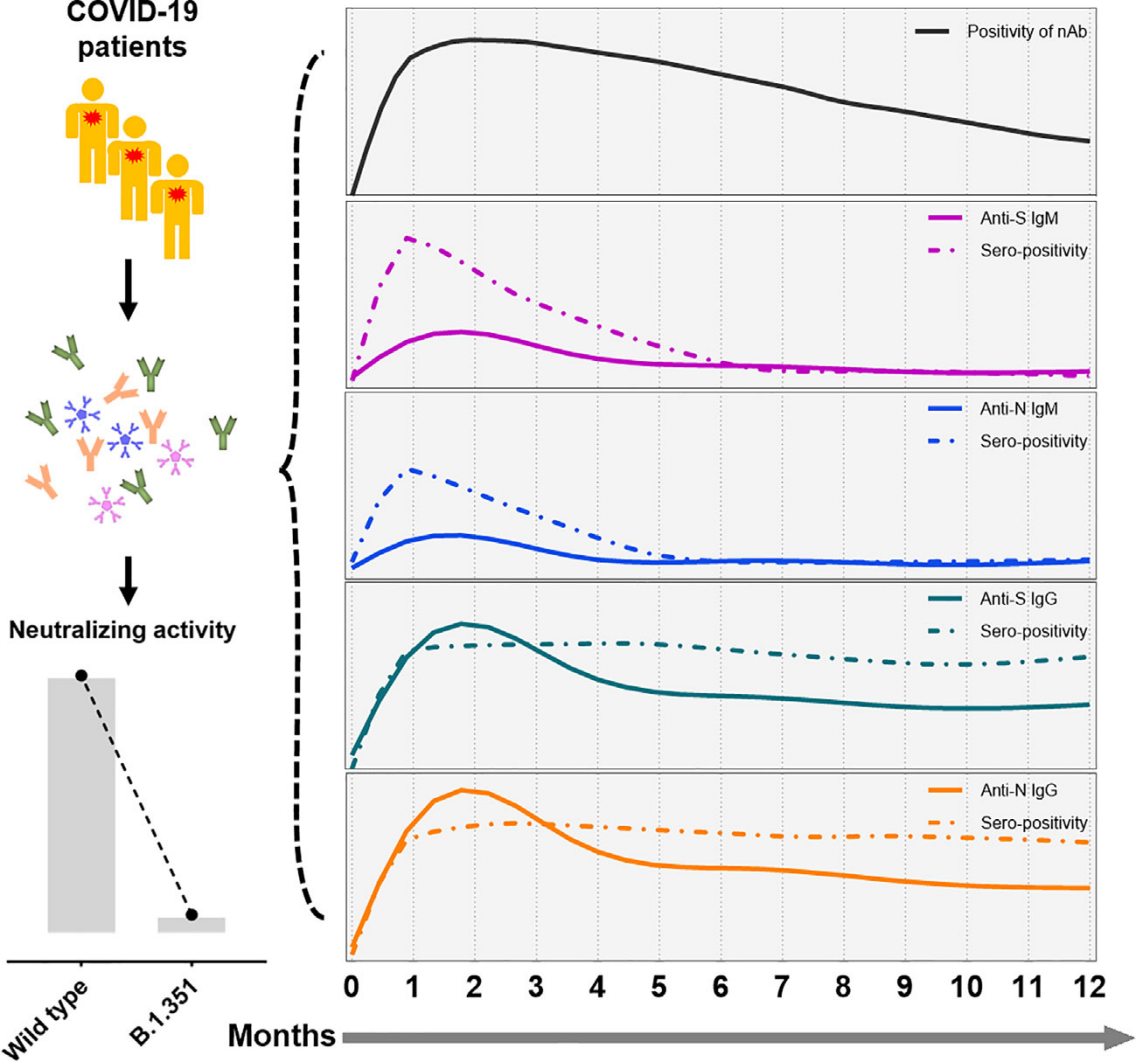
A schematic representation of SARS-CoV-2 antibody responses following symptomatic infection. In summary, humoral immune responses against SARS-CoV-2 decline over time in convalescent COVID-19 patients. While IgM antibody titers decline rapidly, SARS-CoV-2-specific titers of IgG showed a slower decay rate. Furthermore, minor neutralization against the VOC B.1.351 were detectable in convalescent COVID-19 patients compared to wild type SARS-CoV-2. The x-axis indicates the timeline following the onset of symptoms. The continuous curves depict the actual response (e.g., CLIA reactivity), while the dashed lines show the percentage of positivity for neutralizing antibodies (black), and anti-S IgM (purple), anti-N IgM (blue), anti-S IgG (green), and anti-N IgG (yellow line).

Dan et al. recently showed that SARS-CoV-2-specific IgG antibodies can be maintained for up to 8 months (20), but 93% of the patients in their study were never hospitalized. Little information is available about the long-term kinetics of antibodies in symptomatic patients, especially those with severe disease. Our study enrolled convalescent patients following symptomatic infection and found that SARS-CoV-2 specific IgG antibody and nAb can in principle persisted for up to one year. We found that the titers of antibody decline at a slower decay rate 6 months after symptom onset, indicating a rather typical antiviral immune response in which an early expansion phase is followed by a contraction phase that carries over into a longer memory phase. A schematic representation of the longitudinal SARS-CoV-2 antibody response following natural infection is depicted in **Figure 6**, which displays the declining trends of different virus-specific antibodies and neutralizing activity against different virus strains.

Although the principle presence of SARS-CoV-2-specific antibodies from convalescent patients was maintained for a relatively long time, most nAb titers fell below a titer of 1:160, raising some concerns whether such low levels of nAb would be sufficient to completely prevent re-infections. However, in combination with the knowledge that antibody-positive patients have a significantly reduced risk of reinfection compared with antibody-negative patients (31), it is reassuring to find such a high (~90%) positivity rate for IgG one year after symptom onset.

Our study has some limitations: Firstly, a larger sample size would have been desirable. However, given our intention to probe into long-term humoral immunity of convalescent patients whose infections date back so long, adequate study participants are scarce, hard to find and enrol into clinical studies. Secondly, we did not have the chance to assess cellular immune responses such as memory B and T cells. If sustained immune memory indeed exists, re-exposure of antigens would trigger rapid and robust immune responses to protect the body. Thirdly, given the limitations of available clinical specimens, we were only able to test the neutralization activity against VOC B.1.351. Obviously, it would be of importance to conduct similar studies using a comprehensive panel of VOCs and virus under investigation (VUI), including variants harbouring the E484Q mutation such as B.1.617, which is currently very prevalent in India.

In summary, we found that the majority of the convalescent COVID-19 individuals show detectable SARS-CoV-2-specific IgG one year after symptom onset regardless of the disease severity, which may provide some long-term protection against wt-SARS-CoV-2. Certain concern arises from the finding that the titers of neutralizing antibody were relatively low and were mostly incapable to neutralize the VOC B.1.351. Thus, boosting of the natural infection by vaccination should be considered.

## Data Availability Statement

The original contributions presented in the study are included in the article/**Supplementary Material**, further inquiries can be directed to the corresponding authors.

## Ethics Statement

The studies involving human participants were reviewed and approved by Ethics Committee of Wuhan Union Hospital, Tongji Medical College, Huazhong University of Science and Technology. The patients/participants provided their written informed consent to participate in this study.

## Author Contributions

TX and BL designed the experiments, enrolled patients, and performed experiments, analysed data and wrote the manuscript together - the latter two together with UD and MT. YF, SS, and FD isolated the virus and performed neutralization test. SiL, SuL, HW, HL, BW, BZ, and JW contributed to sample and data collection. JL, ML, DY, UD, MT, XZ and FD conceived the study, interpreted the data, and revised the manuscript. All authors contributed to the article and approved the submitted version.

## Funding

This work was supported by the National Science and Technology Major Project for Infectious Diseases of China (2018ZX10302206, 2018ZX10723203, and 2017ZX10304402-002-005); the Fundamental Research Funds for the Central Universities (2020kfyXGYJ016 and 2020kfyXGYJ028); the Applied Basic and Frontier Technology Research Project of Wuhan (2020020601012233) the Innovation Team Project of Health Commission of Hubei Province [WJ2019C003]; the Tongji-Rongcheng Center for Biomedicine, Huazhong University of Science and Technology, and the Medical Faculty of the University of Duisburg-Essen. MT receives funding from the Stiftung Universitätsmedizin Essen (University Hospital Essen, Germany), the Kulturstiftung Essen, the Volkswagenstiftung (through grant #99078), and the Deutsche Forschungsgemeinschaft (through grants RTG1949/2, TR1208/1-1, and TR1208/2-1).

## Conflict of Interest

The authors declare that the research was conducted in the absence of any commercial or financial relationships that could be construed as a potential conflict of interest.
