# Supplementary material for: Declining Levels of Neutralizing Antibodies Against SARS-CoV-2 in Convalescent COVID-19 Patients One Year Post Symptom Onset
Source: Front Immunol. 2021 Jun 16;12:708523. doi: 10.3389/fimmu.2021.708523 (PMC8242354; doi:10.3389/fimmu.2021.708523)
Supplement: Supplementary file 1 [file DataSheet_1.pdf]

Supplementary Material

Table S1. Sensitivity and Specificity of CLIA assays for Diagnosis of SARS-CoV-2 specific antibodies.

	Sensitivity			Specificity		
	Negative	Positive	%	Negative	Positive	%
**Total IgM**	9	136	93.80%	95	1	98.96%
Anti-S IgM	28	117	80.69%	95	1	98.96%
Anti-N IgM	63	82	56.55%	96	0	100.00%
**Total IgG**	4	141	97.24%	94	2	97.92%
Anti-S IgG	10	135	93.10%	95	1	98.96%
Anti-N IgG	6	139	95.86%	95	1	98.96%

Abbreviations: CLIA, capture chemiluminescence immunoassays; The diagnostic value of sensitivity was evaluated in 96 healthy individuals without COVID-19. The diagnostic value of specificity was evaluated in 145 confirmed COVID-19 patients, with disease course ≥11 days and ＜30days.

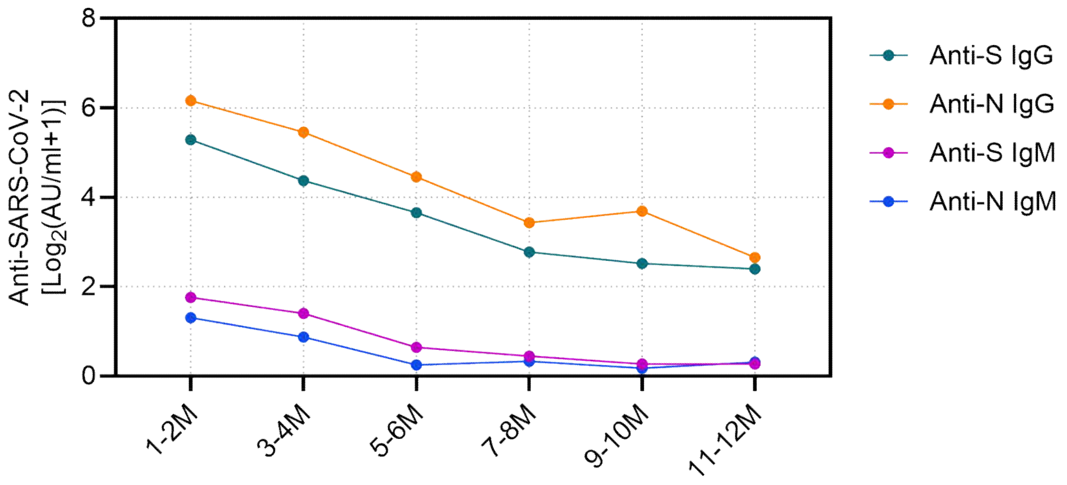

Figure S1. A line chart showing the declining trend of antibodies. A total of 162 samples from 76 patients against different SARS-CoV-2 proteins in longitudinal time points. The dots with different colors represent antibody titers of IgM and IgG recognizing the nucleocapsid (N) protein or the receptor binding domain (RBD) of the spike protein (S).
